# Physiotherapy students’ perceptions of managing patients with mental illness: A qualitative exploration at a South African university

**DOI:** 10.4102/sajp.v82i1.2355

**Published:** 2026-07-16

**Authors:** Maylene Salo, Farhana Karachi, Danelle Hess, Shamila Gamiet

**Affiliations:** 1 Department of Physiotherapy, Faculty of Community and Health Sciences, University of the Western Cape, Bellville, South Africa; 2 Interprofessional Education Unit, Faculty of Community and Health Sciences, University of the Western Cape, Bellville, South Africa

**Keywords:** clinical practice, management, mental health, mental illness, perceptions, physiotherapy

## Abstract

**Background:**

Mental illness (MI) is a leading cause of global ill health and disability, with nearly half of the population affected by mental health (MH) conditions at some point in their lives. Consequently, all healthcare professionals, including physiotherapy students, will inevitably encounter patients with MI in clinical practice. However, their preparedness and ability to manage these patients remain unclear.

**Objectives:**

To explore physiotherapy students’ perceptions of their preparedness and ability to manage patients with co-existing (undiagnosed or known) MH conditions alongside a primary physical health presentation in clinical practice.

**Method:**

A qualitative, exploratory, descriptive design was employed. Sixteen fourth-year physiotherapy students from the University of the Western Cape (mean age 22.8 ± 1.32 years) participated in semi-structured individual interviews conducted either face-to-face or online. Data were thematically analysed using Braun and Clarke’s six-phase method.

**Results:**

Four themes emerged from the data: (1) Evolving understandings of MI: knowledge, sources, and lived experiences; (2) Negotiating care within personal and clinical realities; (3) Insufficient alignment between theory and practice; and (4) Making sense of physiotherapy’s contribution to MH care.

**Conclusion:**

The findings highlight the need to strengthen both theoretical and practical components of MH curricula and training within undergraduate physiotherapy programmes.

**Clinical implications:**

Improved curricular integration may enhance students’ preparedness, ability, confidence and competence when managing patients with MI, while supporting more holistic and effective patient care.

## Introduction

Mental illness (MI) represents a major public health challenge worldwide, contributing significantly to the overall burden of disease and disability. According to global estimates, nearly half of the population will experience an MI at some point in their lives, making it one of the leading causes of ill health (World Health Organization 2022). The implications of this are far-reaching and extend beyond individuals affected by MI to their families, communities, and have an impact on healthcare provision. Considering this, all healthcare professionals, regardless of their area of practice, are increasingly likely to encounter patients living with MI in clinical practice (Gyllensten et al. 2011; Vancampfort et al. 2018). Physiotherapists, traditionally recognised for their role in the physical rehabilitation of patients, are no exception.

Physiotherapists are frequently required to treat patients whose physical conditions are accompanied or complicated by co-existing (undiagnosed or known) mental health (MH) concerns. Anxiety, depression, and stress-related conditions, for instance, may influence recovery trajectories in musculoskeletal rehabilitation, adherence to exercise programmes, and overall health outcomes (Stults-Kolehmainen & Sinha 2014). Therefore, physiotherapists require not only the skills to treat patients’ physical conditions such as respiratory, critical injury and neurological conditions (Liu et al. 2023; Saida et al. 2025; Yohannes & Alexopoulos 2014), but also the competence, knowledge, attitudes, and behaviours that allow them to manage patients holistically by integrating the physical and psychological dimensions of care (Hooblaul, Cobbing & Daniels 2020). Globally, there is increasing recognition of the need to integrate MH competencies into physiotherapy curricula. Initiatives in some countries have focused on embedding MH modules, interprofessional training, or clinical placements in psychiatric settings (Connaughton & Gibson 2016b; Gyllensten et al. 2011; Hooblaul et al. 2020; Probst & Peuskens 2010). These efforts aim to ensure that graduates enter practice with the ability to support patients whose recovery is shaped by psychosocial as well as physical factors. However, research suggests that physiotherapists often feel inadequately prepared for this aspect of their professional role (Connaughton & Gibson 2016b; Hooblaul et al. 2023). This lack of preparedness is partly rooted in physiotherapy education, where MH is often given limited attention compared with other areas of practice. Students, in particular, may feel uncertain about their ability to appropriately manage patients with MI. Inadequate training, combined with negative societal attitudes and stigma, has been found to undermine students’ confidence and limit the quality of care they deliver (Gunduza, Lord & Keller 2023). Therefore, the educational preparation of physiotherapy students to manage patients with MI emerges as a critical area for investigation and development. In the South African context, this is also a significant challenge. With one in every six South Africans currently struggling with an MI, a need for comprehensive and holistic care has become essential (Booysen, Mahe-Poyo & Grant 2021).

Physiotherapists, as part of the country’s broader rehabilitation workforce, occupy an important position in addressing these challenges. However, their preparedness to manage patients with MI has not been well documented. Research suggests that physiotherapy education in South Africa has historically prioritised biomedical aspects of care, with limited emphasis on MH (Gunduza et al. 2023). Students on clinical rotations may therefore encounter patients with co-existing MI while feeling inadequately equipped to manage their needs. This lack of preparedness is problematic for both patient outcomes and students’ professional development. Furthermore, the lack of preparedness can contribute to missed or delayed identification of psychological symptoms, suboptimal patient–provider communication, and fragmented care, ultimately compromising patient outcomes and continuity of care (Hooblaul et al. 2023). At the same time, students may experience uncertainty or reduced confidence in their clinical decision-making, which can hinder their professional growth and limit their ability to provide holistic, patient-centred care. From a patient care perspective, insufficient confidence and limited understanding of MI may negatively influence therapeutic engagement, communication, adherence to treatment, and overall rehabilitation outcomes. This is particularly significant where psychological factors significantly shape recovery trajectories (Stults-Kolehmainen & Sinha 2014; Vancampfort et al. 2018). From an educational perspective, students who feel unprepared may experience increased anxiety, uncertainty, and avoidance behaviours in clinical encounters. This can hinder the development of clinical reasoning, professional identity, and confidence in managing complex patients (Connaughton & Gibson 2016b; Gunduza et al. 2023). Addressing these challenges through curriculum transformation has the potential to strengthen both educational and health system outcomes. Integrating MH competencies more explicitly within physiotherapy training may enhance students’ preparedness and confidence. Opportunities for applied learning and supported clinical exposure may further strengthen their ability to deliver holistic, patient-centred care. In turn, this may contribute to improved patient engagement, more effective rehabilitation, and better overall quality of life for individuals living with MI, particularly within resource-constrained health systems where physiotherapists play a key role in multidisciplinary care.

Physiotherapists working in MH care support individuals and groups across health promotion, prevention, treatment, and rehabilitation within a biopsychosocial framework. They utilise movement, physical activity, and therapeutic relationships to enhance both physical and mental wellbeing. This is underpinned by scientific evidence and contributes to multidisciplinary and interprofessional care to address the complex needs associated with MH (Hooblaul et al. 2023). Managing patients with co-existing MI, therefore, requires physiotherapy students to extend beyond traditional physical rehabilitation skills and develop competencies in biopsychosocial care, communication, and collaborative practice. A scoping review by Hooblaul et al. (2023) identified key theoretical competencies, including recognition of signs, symptoms, and underlying pathophysiology of MH conditions; knowledge of psychiatric medications and their side effects; and a clear understanding of the physiotherapist’s scope of practice. The review further emphasised the importance of incorporating physiotherapy-specific interventions, understanding the roles of multidisciplinary team members, and appropriate referral pathways into physiotherapy education.

Within the context of this study, MH content encompassing both theoretical and clinical competencies is not presented as a clearly defined or formally structured component of the undergraduate physiotherapy curriculum, both within the study setting and more broadly across South African programmes (Hooblaul et al. 2024). Instead, such content is addressed in a limited and integrated manner. Although the Health Professions Council of South Africa outlines minimum standards for undergraduate physiotherapy training, including prescribed credits across core domains (Health Professions Council of South Africa [HPCSA] 2023), the extent to which MH competencies are explicitly defined and consistently embedded remains unclear. Consequently, the breadth and depth of MH-related competencies are neither clearly articulated nor uniformly integrated within South African physiotherapy curricula (Hooblaul et al. 2024). In this study, the physiotherapy curriculum comprises a single psychology module in both the first and second years. However, the content of these modules provides limited coverage of MH theory, practical competencies, and application to clinical practice. These gaps in the physiotherapy MH curriculum provided the impetus for the present study, which aimed to explore this cohort’s perceptions of their preparedness and ability to manage patients with co-existing MI in clinical practice.

Despite the relevance of this topic, few studies have explored the perceptions of physiotherapy students in South Africa regarding their readiness to manage patients with MI in clinical practice. Understanding physiotherapy students’ experiences and perspectives is critical for identifying educational gaps and informing curriculum reform in the profession. Therefore, the aim of this paper is to explore and describe undergraduate physiotherapy students’ perceptions of their preparedness and ability to manage patients with co-existing (undiagnosed or known) MH conditions alongside a primary physical health presentation in clinical practice. Preparedness is examined through students’ self-reported knowledge and understanding, attitudes and behaviours, clinical experiences, and their perceptions of facilitators and barriers to care.

## Method

### Study design

A qualitative, exploratory, descriptive design was employed for this study. This approach was underpinned by an interpretivist–constructivist paradigm, which assumes that reality is socially constructed and that individuals make meaning of their experiences within specific social and educational contexts. A qualitative design was therefore appropriate, as the study sought to explore physiotherapy students’ subjective perceptions, experiences, and interpretations of managing patients with co-existing (undiagnosed and known) MI in clinical practice.

An exploratory design was selected, given the limited research available on this topic within the South African physiotherapy context, allowing for an open and in-depth investigation without imposing predefined assumptions or categories. The descriptive component enabled a detailed and contextually grounded account of participants’ experiences, ensuring that their perceptions were represented as closely as possible to how they were expressed. This combination of exploratory and descriptive approaches facilitated a nuanced understanding of students’ perceptions of their preparedness and ability to manage patients with co-existing MI (Tenny, Brannan & Brannan 2022).

### Participant sampling and recruitment strategy

The target population for this study included all physiotherapy students registered in their fourth year of study in 2023 and 2024, respectively (*n* = 143). This population was exposed to clinical training during their third and fourth years of study at a University in the Western Cape, South Africa. Participants would likely have encountered patients with MI or underlying MI during their clinical rotations. The study population was also able to provide valuable insight into the mostly theoretical component taught in the first and second years of the undergraduate physiotherapy programme. The 2023 cohort had completed both their third- and fourth-year clinical training rotations. The 2024 cohort had completed their full third year and at least two fourth-year clinical training rotations. A total inclusive purposive sampling method was used, with all 143 students invited to participate in the study. Inclusion criteria for this study were: All final-year physiotherapy students at the University of the Western Cape who had completed at least two clinical rotations in their fourth year, in addition to their third-year clinical experience. No exclusion criteria were applied within the defined study population.

### Data collection procedure

All fourth-year physiotherapy students were invited to participate in the study via an email sent to them by their clinical coordinator, informing them of the nature and purpose of the study through an information sheet. Sixteen (*n* = 16) students agreed to participate in either face-to-face or online individual audiotaped interviews at a time and place convenient to them and the researcher. The consent forms were completed online and emailed to the researcher, who then booked the interview time slots. A semi-structured interview guide (Online Appendix 1) was developed by the researcher to address the study objectives and collect relevant data. Given the sensitivity of the topic and the potential discomfort of discussing MI among peers, one-on-one interviews were conducted. The interview questions were informed by existing literature and further discussed with the research supervisors for ratification to align with the objectives of the study. The Attitudes Towards Psychiatry 30-item questionnaire (Burra et al. 1982) served as a reference for formulating interview guide questions. The interview guide covered students’ knowledge, feelings about people with MI, their management of patients with MI, and factors affecting their management of patients with MI. It also explored the role of the physiotherapist in managing patients with MI and additional information that may assist with the management of patients with MI. For each participant, age and gender were documented. A pilot interview was conducted with one third-year physiotherapy student to assess the clarity of the questions, their alignment with the study objectives, and the interview duration. The questions were found to be clear and appropriate. Data from the pilot were excluded from the final analysis as the pilot participant had only completed their third year of study. Including the above would have contrasted with other participants, who had all completed their third year of study and a minimum of two clinical rotations in their fourth year. The use of only one student to pilot the interview guide could be seen as a minor limitation, as it may explain the lack of changes to the guide. However, evidence states that while methodological texts recommend the use of pilot studies in qualitative research, there is a lack of reported research focusing on how to conduct such pilot studies. Harding (2013) states that it is not relatively obvious as to the need for qualitative interviews to be piloted, as the interviews usually progress and the quality of the interview guide improves with each interview. However, Harding (2013) mentioned that piloting the interview questions and adjusting the interview guide accordingly is notably helpful before embarking on a major study. The interview guide was also piloted for content validity. The research supervisor (S.G.) was provided with the interview guide and was requested to provide input on whether the questions were appropriate for answering the research question, aim, and objectives of the study. The research supervisor noted that all questions in the interview guide aligned with answering the objectives of the study, and therefore, no changes were made. Sixteen individual interviews were conducted. Four interviews were conducted face-to-face, and 12 were conducted online via the Google Meet platform. The interviews lasted between 25 min and 45 min each. The conversational, open-ended format allowed for in-depth exploration, with follow-up questions and field notes used to capture nonverbal cues. Data saturation was determined when no substantially new codes, concepts, or patterns emerged from subsequent interviews. Although interviews were not fully coded immediately after each interview, transcription, familiarisation with the data, and preliminary identification of recurring concepts occurred alongside the data collection process. By the 16th interview, sufficient repetition and thematic redundancy were observed, indicating that data saturation had been reached. This approach is supported by Lincoln and Guba (1985), who proposed the discontinuation of sampling once no new information is forthcoming.

### Data management and analysis

The online interviews were audio-recorded with the participants’ consent and transcribed verbatim by the researcher by utilising the Google Meet (Google LLC, Mountain View, CA, United States [US]) recording function. Face-to-face interviews were recorded using a password-protected mobile device. All audio recordings were securely managed during transcription using Otter.ai (Otter.ai, Inc., Mountain View, CA, US), a password-protected and encrypted third-party transcription service that complies with data protection and confidentiality standards. All files were anonymised prior to upload, and access to the platform was restricted to the primary researcher. To ensure transcription accuracy, each transcript was checked against the original audio recording by the researcher, with corrections made where necessary. This process involved repeated listening to the recordings to verify completeness and fidelity to participants’ responses. The researcher then reviewed and cross-checked each transcript carefully against the audio recordings to ensure accuracy. Transcripts were verified by the researcher and research supervisors by reading through them multiple times to ensure accuracy. The data were analysed using Braun and Clarke’s six-phase thematic analysis, employing manual coding, making use of Microsoft Word as a tool (Braun & Clarke 2019). Manual coding was deemed appropriate for this study as it enabled deeper engagement with the data and allowed subtle contextual and cultural means to be recognised (Shahid 2025). An inductive approach was followed to generate codes. This approach allowed for codes to develop in the course of the analysis (Bingham 2023), with themes fluidly developing through the analytical process as per Braun and Clarke (2019). Initial themes were formed and named. After further review by the researcher and the research supervisors, the themes were reviewed and refined, leading to the formation of subthemes. Further review of themes and subthemes resulted in the researcher identifying prevalent key ideas of the data by presenting the final themes and subthemes in a clear and concise manner.

### Quality criteria

For trustworthiness, the researcher used Lincoln and Guba’s (1985) criteria as a means to evaluate the rigour and quality of the research. This framework includes credibility, transferability, dependability, and conformability. Credibility was supported by the process of member checking. This process provides credibility to the data through sending interview transcripts to the participants to confirm accuracy and/or to provide feedback (McKim 2023). Transferability was ensured by logging a detailed description of the findings of the study, from the events leading up to data collection, the recruitment processes, and the method of data analysis employed (Shenton 2004). The meticulous and detailed documentation of all the research procedures, analysis, and interpretation provided dependability. The researcher is of the opinion that these steps would ensure transparency and reproducibility of the research. To establish conformability, the researcher leveraged reflexivity to identify, acknowledge, and eliminate her own biases. To maintain reflexivity, the researcher remained aware, acknowledged, and reflected on the ways in which her position as a clinical educator at the time of the data analysis might have influenced the research process (Olmos-Vega et al. 2022). In recognising this, the researcher was intentional about ensuring that participants’ participation in the study was voluntary and clarified that their involvement in the study would not unduly influence participants’ progression, evaluation, or clinical assessment in any way. Reflexivity also informed the manner in which the interviews were conducted, with the researcher adopting a supportive and non-judgemental stance to promote participant comfort and emotional wellbeing. Confidentiality was prioritised, and member checking as well as supervisory review were employed to reduce imperative bias.

### Ethical considerations

Ethical clearance to conduct this study was obtained from the University of the Western Cape Humanities and Social Science Research Ethics Committee (No. HS23/9/4), with institutional permission granted to conduct the study. All procedures complied with relevant ethical standards. Participation was voluntary, and participants could withdraw at any time without consequence. Written informed consent was obtained from all participants. Anonymity and confidentiality were ensured through the use of pseudonyms and anonymised transcripts, which were securely stored in a password-protected Google Drive folder accessible only to the primary researcher and supervisors (Maylene Salo, Shamila Gamiet and Danelle Hess). Audio-recorded interviews on the password-protected mobile device were saved to the password-protected Google Drive folder and then deleted from the device. Data handling complied with the *Protection of Personal Information Act No. 4 of 2013* (Government Gazette South Africa 2013). Given the sensitive nature of the study, participants were informed of the available counselling support through the Centre for Student Support Services.

## Results

Sixteen final-year physiotherapy students participated in the study (*n* = 16). None were repeat students, and all had completed a comparable number of clinical rotations. The mean age was 22.8 ± 1.32 years, and most participants were female (73%, *n* = 11). Four themes emerged from the data, namely: (1) Evolving understandings of MI: Knowledge, sources, and lived experiences; (2) Negotiating care within personal and clinical realities; (3) Insufficient alignment between theory and practice; and (4) Making sense of physiotherapy’s contribution to MH care.

### Theme 1: Evolving understandings of mental illness: Knowledge, sources, and lived experiences

‘I don’t know if this is too personal, but I self, dealt with depression growing up.’ (PI, 22 years old, Female)

Participants demonstrated varying levels of depth of knowledge and understanding of MI, shaped by a mix of formal teaching, informal exposure, and deeply personal experiences. While some offered biologically oriented descriptions, linking MI to cognitive factors, hormonal imbalance, substance use, or changes in brain structure, others framed MI more broadly as shifts in mood, behaviour, social interaction, or cognitive functioning. For many, their knowledge centred primarily on commonly discussed conditions such as depression and anxiety, highlighting what felt familiar:

‘In school, they teach us, what they teach us, the types of MI, like they would speak about anxiety, and depression, but I don’t think they go in depth …’ (P[M], 22 years old, Female)

‘What I, what I know about mental illnesses is that people some people are born with it, some acquire it from substance abuse.’ (P[A], 25 years old, Female)

‘So, in terms of what I know about MI, I just know it’s a broad term for any change that occurs. Could be in someone’s mood with regards to their social interactions, their cognitive ability and state. So, any change in that, that could be as a result of a pathology, or just imbalance of hormones.’ (P[B], 21 years old, Male)

The physiotherapy curriculum alone did not enhance the students’ understanding of MI. Participants described a patchwork of learning experiences beginning as early as high school Life Orientation, which introduced the concept but was often experienced as superficial or incomplete. University psychology modules added theoretical grounding, yet these were described as limited in scope, easily forgotten over time, and not consistently reinforced across the physiotherapy curriculum:

‘So, the first time I learned about MI was in high school, 2018 during our LO [*Life Orientation*] classes, but then they didn’t go into depth about what MI is actually, and what causes MI. And then in first year, we had a module, psychology.’ (P[N], 22 years old, Female)

‘But where you learn about other mental health illnesses is just like one module that you learn, you passed and then you sort of forgot everything after that.’ (P[O], 21 years old, Female)

Other sources of knowledge acquisition of MI included podcasts, social media, online searches, and public awareness campaigns. These sources filled knowledge gaps and shaped the students’ perceptions and interpretation of MI in everyday life:

‘That’s where I learned more about MI and how it affects people. I also learned about it, on, on podcasts, and also on Miss SA [*South Africa*] 2021.’ (P[N], 22 years old, Female)

‘And then I think, social media has a big effect on my exposure to MI. And then when I see things on there that, like, makes me go look for more information.’ (P[F], 21 years old, Male)

Personal experience surfaced as one of the most powerful contributors to students’ understanding of MI. Several participants spoke openly about their own histories with depression or exposure to peers and family members living with MI:

‘I don’t know if this is too personal, but I self, dealt with depression growing up. Like from a young age, like from 12 years old, I’ve dealt with that … I came to a state where I didn’t want to be alive anymore.’ (P[I], 21 years old, Female)

‘I have actually known about MI, illness, sorry, for as long as I can remember. Because my uncle has a MI. So I’ve sort of grown up with it all my life.’ (P[B], 21 years old, Male)

### Theme 2: Negotiating care within personal and clinical realities

‘… I am a bit more patient with my patients compared to maybe other students.’ (P[F], 21 years old, Male)

The quote below reflects the theme of negotiating care within personal and clinical realities by illustrating how student physiotherapists must balance their professional responsibilities with immediate concerns for personal safety and environmental constraints when managing patients with underlying MI, highlighting the complex, often unpredictable realities that shape care decisions in clinical settings:

‘And then he drew the curtain because he was talking, but you couldn’t make sense of what he was saying. And then he pushed me and I bumped into those shelves next to the bed. I was so scared because I’m thinking of what would have happened if it was a hospital where you take a patient into a separate room?’ (P[L], 21 years old, Female)

Students’ management of patients with MI was further shaped by a combination of personal history, broader societal beliefs and clinical exposure. Personal MH experiences played a significant role. For some students, unresolved challenges created discomfort or fear of being triggered while managing patients with MI, while others felt that lived experience strengthened their empathy, patience, and ability to connect with patients:

‘Anxiety, also, because I think I also have like, anxiety issues. So, I feel like if I were to treat you and you also suffered from anxiety, it would not, it would trigger me as well.’ (P[J], 21 years old, Female)

‘I think it contributes to the feeling empathetic part, in that I am a bit more patient with my patients compared to maybe other students.’ (P[F], 21 years old, Male)

Stigma formed an additional layer of influence in managing patients with underlying MI. Many students entered university with stigmatised beliefs shaped by community narratives associating MI with danger, instability, or supernatural causes. Education and clinical exposure helped shift these perceptions:

‘I think my perception around MI was more, I can say, affected by stigma, like in society before you come to university, before you have knowledge about things. There is this stigma that says, like, mentally ill people are emotionally unstable, or they are unwell, so they’re just mad.’ (P[G], 21 years old, Female)

‘So, you know, back home, people have this thing of: “No, he is bewitched”.’ (P[A], 25 years old, Female)

‘… I used to be scared of people with MI and never wanted to associate myself with them. Because I believed somehow they posed a danger to my life when I didn’t know any better.’ (P[N], 22 years old, Female)

Clinical exposure further shaped confidence and behaviour and assisted in developing competency skills in the management of patients with co-existing MI. Supportive interactions and opportunities to build rapport enhanced students’ sense of capability, whereas unpredictable or emotionally intense encounters exposed perceived gaps in training and contributed to feelings of uncertainty or fear. Collectively, these experiences highlighted the importance of clearer guidance and more robust preparation to better support students in managing patients with MI within real-world clinical settings. Students also described drawing on their own interpersonal qualities to navigate these emotionally complex encounters, particularly through listening and maintaining a non-judgemental stance, as reflected in the following quotes:

‘I’m very patient and I listen … they just need someone to listen to them. Someone who’s not biased …’ (P[F], 21 years old, Male)

‘She didn’t understand why she was there, and I found it very difficult to treat her. I also didn’t have enough background on treating like, like, how do you treat the patient like that?’ (P[I], 21 years old, Female)

### Theme 3: Insufficient alignment between theory and practice

‘Because if I try to think back of like, all the lectures and stuff that we had, it didn’t really prepare me to treat a patient with MI.’ (P[I], 21 years old, Female)

Students consistently described a gap between what their training prepared them to do and what they were required to manage upon entering the clinical platform. Early psychology content offered some theoretical grounding, the material was remembered as broad, and insufficiently linked to real clinical expectations. In addition, due to psychology modules being taught in their first and second years of study, by the time students encountered patients presenting with MI, much of this content had faded or felt too abstract to guide assessment, or safe patient interaction:

‘So, I don’t recall at any point, during my studies, I was given a thorough lecture, which I thought is quite necessary, on how to possibly approach a patient with some sort of, sort of MI.’ (P[B], 21 years old, Male)

‘But where you learn about other mental health illnesses is just like one module that you learn, you passed and then you sort of forgot everything after that.’ (P[O], 21 years old, Female)

‘If I can remember cause first and second year was so long ago, but I honestly don’t remember learning anything about MI … Because if I try to think back of like, all the lectures and stuff that we had, it didn’t really prepare me to treat a patient with MI.’ (P[I], 21 years old, Female)

Across interviews, students expressed a need for more practical, applied preparation. They wanted exposure to real-world scenarios, structured case discussions, opportunities to learn from MH professionals, and clearer guidance from lecturers with relevant clinical experience. In the absence of this, many relied on ad hoc learning from clinicians during rotations, highlighting the reliance on informal rather than curricular preparation:

‘I think I would have liked more background info on the different mental illnesses and maybe how we can, how like be aware of it when we treat patients. … like our lecturers’ experience, maybe would have helped, like how to deal with a patient … And I think I learned more from maybe the clinicians at the hospitals where I worked at, because they will maybe had more experience treating patients like that.’ (P[I], 21 years old, Female)

‘… maybe invited people in the psychiatric, someone with psychiatry to maybe come to us and explain in further details.’ (P[A], 25 years old, Female)

Students also identified the timing and scope of ethics training as misaligned with clinical reality. As ethics is introduced only in the fourth year, many students had already faced complex MH-related dilemmas during earlier clinical rotations without the tools to navigate them. While ethical discussions sometimes referenced MI, students felt they lacked the depth and specificity required for meaningful application:

‘… we do have an ethics module where this type of conversations are spoken about in class, but not as in depth as I would actually like it because it is, it’s more like discussions and you’re sharing an experience, you know, an opinion.’ (P[F], 21 years old, Male)

‘But we’re never really equipped, because even with ethics, we’re only taught ethics when we’re doing our like fourth year, and the damage honestly has been done by then.’ (P[K], 22 years old, Male)

### Theme 4: Making sense of physiotherapy’s contribution to mental health care

‘I don’t know what is it. Our role.’ (P[I], 21 years old, Female)

Students recognised that physiotherapists can play a meaningful part in supporting patients with MI, but they also acknowledged that this role is not clearly defined. Many participants viewed physiotherapists as well placed to contribute to mental wellbeing through motivation, education, active listening, and sustained therapeutic rehabilitation. Because physiotherapists often spend more time with patients than other health professionals, students felt they (physiotherapists) were uniquely positioned to build rapport, pick up on emotional shifts, and support both patients and families:

‘They [*physiotherapists*] empower the individual, you know, they motivate you to actually want to be independent, as much as possible on your own …’ (P[H], 21 years old, Female)

At the same time, students were aware of the limits of their scope of practice and expressed uncertainty about how far their responsibilities extended, with some even expressing not knowing what the physiotherapist’s role is at all. Limited exposure to MH within their training contributed to this ambiguity, leaving some unsure of what physiotherapy ‘should’ look like when MI complicates rehabilitation. For these students, their contribution often defaulted to reassurance, empathy, and referral to MH specialists when concerns exceeded their competence:

‘And I do feel like there’s only so much we as physios can do.’ (P[D], 21 years old, Female)

‘Just trying to be there for them [*patients*], to be empathetic. I don’t know what is it. Our role.’ (P[I], 21 years old, Female)

In summary, the results highlight variability in students’ perceptions of their preparedness to manage patients with co-existing MI, shaped by prior exposure, perceptions of their competence, and the level of support available within academic and clinical settings. While the themes are presented separately, they represent interconnected aspects of students’ experiences rather than a linear or hierarchical process.

## Discussion

This study explored undergraduate final-year physiotherapy students’ perceptions of their preparedness and ability to manage patients with (undiagnosed or known) MH conditions alongside a primary physical health presentation in clinical practice. The findings showed that students’ knowledge of MI was shaped by fragmented curricular exposure, personal lived experience, and informal learning rather than structured physiotherapy training. This aligns with previous findings suggesting that undergraduate physiotherapy education often provides inconsistent and limited MH preparation (Booysen et al. 2021). Students reported challenges in negotiating care within complex personal, social, and clinical realities, compounded by stigma, safety concerns, and limited confidence and preparedness. Dandridge et al. (2014) highlight similar findings where undergraduate physiotherapy students reported feelings of underpreparedness, limited confidence, and safety concerns. Overall, a clear misalignment between theoretical preparation and clinical demands emerged, alongside uncertainty about physiotherapy’s role in MH care, highlighting the need for more integrated, applied, and scaffolded MH education within physiotherapy curricula (Andrew et al. 2019). The latter findings are supported by authors who have reported an increasing recognition of the need to integrate MH competencies into physiotherapy curricula, as well as initiatives by some countries that have focused on embedding MH modules in their curricula, including interprofessional training or clinical placements in psychiatric settings (Connaughton & Gibson 2016a; Gyllensten et al. 2011; Hooblaul et al. 2020; Probst & Peuskens 2010).

The current study further illustrates the uneven ways in which the undergraduate final year physiotherapy students come to understand MI, such as through personal experiences, social media and fragmented forms of formal and informal training. Similar findings have been reported internationally. Barradell et al. (2025) found that students’ knowledge appeared to be shaped by personal experiences, informal learning, and fragmented curricular exposure, resulting in partial understanding that did not consistently translate into clinical reasoning. Furthermore, anxiety and depression were the most commonly recognised MH conditions by the cohort in this study. This familiarity with anxiety and depression risks narrowing students’ conceptualisation of MI and overlooks its broader spectrum, including severe and enduring MI. These findings are mirrored by Gustavson et al. (2018) and Naser et al. (2021), who also report that students tend to feel more comfortable managing common mental disorders than complex psychiatric presentations, reflecting limited exposure and confidence in MH practice. These fragmented understandings of MH directly influenced clinical decision-making and patient management as reported by the cohort of students in our study. This also supports the broader literature emphasising the importance of integrating biopsychosocial reasoning throughout physiotherapy education rather than confining MH content to isolated modules (Connaughton & Gibson 2016a; Dandridge et al. 2014; Hooblaul et al. 2023).

Students in this study reported that their management of MI was shaped by personal histories, social attitudes, and clinical realities. Personal MH experiences sometimes enhanced empathy towards patients with MI. This aligns with literature suggesting that lived experience can enhance compassionate care and therapeutic engagement (Furnham & Sjökvist 2017). However, these experiences also contributed to feelings of emotional vulnerability and uncertainty in patient care, particularly when students feared being emotionally triggered, as similarly described by Connaughton & Gibson (2016b). Our findings, therefore, highlight the importance of structured opportunities for self-awareness, emotional regulation, and clinical debriefing within health professions education and are supported by Connaughton & Gibson (2016b) and Furnham and Sjökvist (2017). Stigma emerged as both a barrier and a site of transformation for our cohort: while some students entered training with stigmatised beliefs, these shifted through education and clinical exposure. These findings regarding stigma are consistent with findings reported across health professions education (Connaughton & Gibson 2016b; Dandridge et al. 2014). In addition, Abdelmonaem, Osman and Karim (2024) and Furnham and Sjökvist (2017) report that meaningful contact with people experiencing MI, together with guided reflective learning, contributes to reduced stigma and improved attitudes towards MH care among nursing and healthcare professional students. However, our study showed that inconsistent and unstructured exposure to MH in clinical practice produced variable learning experiences, suggesting that meaningful contact alone is insufficient in this context. Instead, our findings support the literature advocating for intentional pedagogical approaches, including facilitated reflection, supervised clinical engagement, and psychologically safe learning environments, to build confidence and reduce stigma in the management of patients with co-existing MI.

A key finding was the misalignment between theoretical teaching and clinical application. Early psychology modules were perceived as broad, decontextualised, and poorly timed relative to clinical exposure, leaving students underprepared when encountering patients with MI. Similar concerns have been identified across physiotherapy and broader health professions education, where foundational MH teaching is often disconnected from discipline-specific clinical application and therefore difficult to translate into practice (Dandridge et al. 2014; Gunduza et al. 2023). Ethics education, introduced late (final year) in the programme, was similarly viewed as insufficient for real-time clinical demands. Students, therefore, entered clinical practice uncertain about their professional role when MI complicated rehabilitation. The latter emphasises the need for longitudinally integrated MH and ethics education, supported by case-based learning, simulation, and interprofessional exposure to strengthen the application of knowledge in clinical practice. Similar recommendations have been as supported by Gunduza et al. (2023), Dandridge et al. (2014), Hooblaul et al. (2023), and Probst and Peuskens (2010).

Students’ perceptions of physiotherapy’s role in MH reflected both recognition and uncertainty. This is evidenced by the absence of explicit HPCSA guidelines regarding the inclusion of MH within undergraduate physiotherapy curricula, potentially contributing to uncertainty around the physiotherapist’s role in MH care and the extent to which MH content should be integrated into training programmes (HPCSA’s Minimum Standards for Training: Physiotherapy 2023). While the students identified meaningful contributions in the management of patients with co-existing MI, including motivation, functional rehabilitation, education, and holistic support, they expressed unclear scope boundaries and limited confidence in application. Furthermore, although students demonstrated awareness of evidence-based interventions such as exercise and breathing strategies, their ability to apply these was constrained by limited curricular integration and inconsistent clinical exposure. Evidence supports physiotherapy interventions such as exercise, physical activity promotion, breathing retraining, and behavioural approaches in improving MH outcomes (Gunduza et al. 2023; Hooblaul et al. 2024; Probst & Skjaerven 2018; Stubbs et al. 2014). Our findings therefore indicate that conceptual knowledge exists but is not consistently translated into practice, highlighting the need for clearer articulation of physiotherapy’s MH role and structured applied learning within multidisciplinary contexts.

In the South African context, the findings of this study are particularly significant. Physiotherapists frequently manage patients whose physical conditions are intertwined with untreated or undiagnosed MI. This often occurs within a resource-constrained public health system characterised by high workloads, limited specialist services, and socio-cultural complexity. In this setting, MH competence is essential rather than supplementary (Hooblaul et al. 2024). The findings of this study call for contextually grounded curriculum reform that integrates MH across training, strengthens clinical scaffolding, and reflects the realities of public-sector practice. Such reform is necessary to bridge the gap between theoretical preparation, clinical expectations, and the evolving professional identity of physiotherapists in South Africa and should be supported by the professional council through clear scope of practice guidelines related to MH in physiotherapy education.

### Study limitations and strengths, and future directions

This study reflects the experiences of final-year undergraduate physiotherapy students from a single South African university, which limits the broader transferability of the findings. Including students from additional institutions may have provided a more diverse representation of curricular structures, clinical exposures, and regional health system pressures. Despite this limitation, the study contributes to physiotherapy education by identifying specific points of misalignment between theoretical teaching and clinical expectations, offering contextually grounded insights relevant to curriculum development. Future research should extend beyond individual perceptions to examine institutional approaches to MH training, compare educational models across South African universities, and identify responsive strategies to meaningfully integrate MH into physiotherapy education and clinical practice.

## Conclusion

The findings highlight the need to strengthen both theoretical and practical components of the MH curriculum and training within undergraduate physiotherapy curricula. A more structured and intentionally integrated MH curriculum within undergraduate physiotherapy training is therefore recommended. Students’ uneven knowledge, limited clinical preparedness, and reliance on personal experience highlight gaps that cannot be addressed through isolated theoretical modules alone. Embedding MH content throughout the curriculum, supported by practical, supervised engagement with patients living with MI, may better align student preparation with clinical realities. This improved curricular integration may enhance students’ preparedness, ability, confidence, and competence when managing patients with MI, while supporting more holistic and effective patient care. Within the South African context, where physiotherapists often work in resource-constrained settings and encounter high MH burdens, such curricula reform is particularly important. Strengthened curricular integration has the potential to produce graduates who are more confident, competent, and equipped to deliver holistic, patient-centred care to this specific patient population.
